# Potential Confounders in the Analysis of Brazilian Adolescent’s Health: A Combination of Machine Learning and Graph Theory

**DOI:** 10.3390/ijerph17010090

**Published:** 2019-12-21

**Authors:** Amanda Yumi Ambriola Oku, Guilherme Augusto Zimeo Morais, Ana Paula Arantes Bueno, André Fujita, João Ricardo Sato

**Affiliations:** 1 Center of Mathematics, Computing and Cognition—Universidade Federal do ABC, Santo André CEP 09210-580, Brazil; 2 Big Data—Hospital Israelita Albert Einstein, São Paulo CEP 05652-900, Brazil; 3 Institute of Mathematics and Statistics—University of São Paulo, São Paulo CEP 05508-090, Brazil

**Keywords:** adolescent, machine-learning, network, graph, public health

## Abstract

The prevalence of health problems during childhood and adolescence is high in developing countries such as Brazil. Social inequality, violence, and malnutrition have strong impact on youth health. To better understand these issues we propose to combine machine-learning methods and graph analysis to build predictive networks applied to the Brazilian National Student Health Survey (PenSE 2015) data, a large dataset that consists of questionnaires filled by the students. By using a combination of gradient boosting machines and centrality hub metric, it was possible to identify potential confounders to be considered when conducting association analyses among variables. The variables were ranked according to their hub centrality to predict the other variables from a directed weighted-graph perspective. The top five ranked confounder variables were “gender”, “oral health care”, “intended education level”, and two variables associated with nutrition habits—“eat while watching TV” and “never eat fast-food”. In conclusion, although causal effects cannot be inferred from the data, we believe that the proposed approach might be a useful tool to obtain novel insights on the association between variables and to identify general factors related to health conditions.

## 1. Introduction

Brazil is the country with the fifth-largest population in the world [1], and as a developing Latin American country, public health care is a major issue for the Ministry of Health [2]. Since 1988, the country adopted a public universal health care system, one of the largest in the world [3,4], which implies several challenges, particularly in primary health. In developing countries, adolescent health is one of the key elements for social progress and economic development, considering that adolescents will become the country’s human resources in the near future.

To better address these issues, the Brazilian Institute of Geography and Statistics (IBGE) and the Ministry of Health (Secretary of Health Vigilance) joined collaborative efforts to map risk factors and adolescent habits across the whole country. They implemented the National Survey of Students’ Health (PeNSE, from the Portuguese abbreviation), a large-scale community-based survey with approximately 130,000 participants. Adolescents are sampled from schools to answer a detailed survey with questions about their health and related matters. This is the largest national survey targeting this population and it is widely used as the empirical foundations to design public policies, which are usually focused on prevention.

Although PeNSE is a very large and rich dataset, most published studies and official reports are descriptive and are based on univariate analyses. The identification of potential confounders is a challenge in health data analysis, mainly when the number of variables is high and the covariance structure is complex. Tackling this problem was the main concern of the current study. In the so-called “ages of Big Data and machine learning”, more sophisticated analytical tools could be used. Particularly, machine learning methods and graph analysis are straightforward frameworks to handle large multidimensional data. Machine learning supervised methods are tools suitable to extract information from many predictive variables to predict another variable of interest [5]. Complementarily, graph analysis is a powerful approach to facilitate the understanding of the relations between a set of variables from a network perspective [6]. Nevertheless, in Brazil, the employment of these algorithms in public health is still scarce, although a few studies have been performed [7,8].

Frequently, public health studies assess the dependence between variables of interest, for example, to identify risk factors for a given condition. A common approach might be to investigate a simple pairwise association between variables, but bivariate analysis might limit the interpretation of the results. There might be further confounding factors or important variables that cannot be properly taken into account within such an approach. A possible solution is to include these additional variables as covariates in linear regressions, but this relies on the assumption of linear relations. Moreover, traditional statistical methods might be unstable in cases of high dimensional data. Here, we used gradient boosting machine, a machine learning approach, as a solution for multivariate analysis that overcome linearity assumptions and high dimensionality problems, while providing the possibility of identifying the important features for a given prediction problem. By using the importance of these features to build a predictive network it is possible to use graph analysis metrics. By definition, hub variables of this network are the variables that are most often considered important by the Gradient Boosting Machines (GBMs), and thus, they are potential confounders or at least variables to be considered when investigating the association between variables.

In the current study, we aimed to identify the most relevant variables associated with adolescence health in Brazil, based on the PeNSE 2015 dataset´s last survey conducted. We focused on the predictive relation between variables from a multivariate perspective, and thus, we used a combination of machine-learning methods and graph analysis to build predictive networks. We hypothesized that variables of socio-demographic characteristics and nutrition would be the most relevant confounders.

## 2. Materials and Methods 

### 2.1. Participants

The sample used in the current study represents students from the 9th grade in Elementary School, depicting the 27 Brazilian federated units, state capitals, and the Federal District. The original survey comprised data from 3160 schools, 4418 classes, with 128,027 students enrolled. Schools with less than 15 students in the 9th grade or only enrolled in night classes (less than 3% of all students) were excluded. In the visited schools, all students enrolled in the 9th grade were individually interviewed using an electronic questionnaire on a smartphone, under the supervision of trained researchers. Data collection was performed between April 2015 and September 2015. The questionnaire was answered by 102,301 students.

The PeNSE survey entails questions about socioeconomic aspects, family context, eating habits, physical activity practice, experimentation and consumption of cigarettes, alcohol and other drugs, sexual and reproductive health, violence, safety, accidents, and use of health services [9]. The 2015 edition was approved by the National Research Ethics Commission (CONEP), which regulates and approves health research involving human beings in the country (no. 1006467). In Brazil, the Child and Adolescent Statute (ECA, Law No. 8.069, of 13 July 1999) requires surveys to have clear objectives and to subsidize policies to protect and provide autonomy to adolescents to take initiatives, such as answering questionnaires. Therefore, students were not obliged to answer sensitive questions and there were no risks to their health. Measures were taken to protect the subject´s identity, to avoid embarrassment, and to make them feel comfortable.

### 2.2. General Procedure

**Figure 1:**
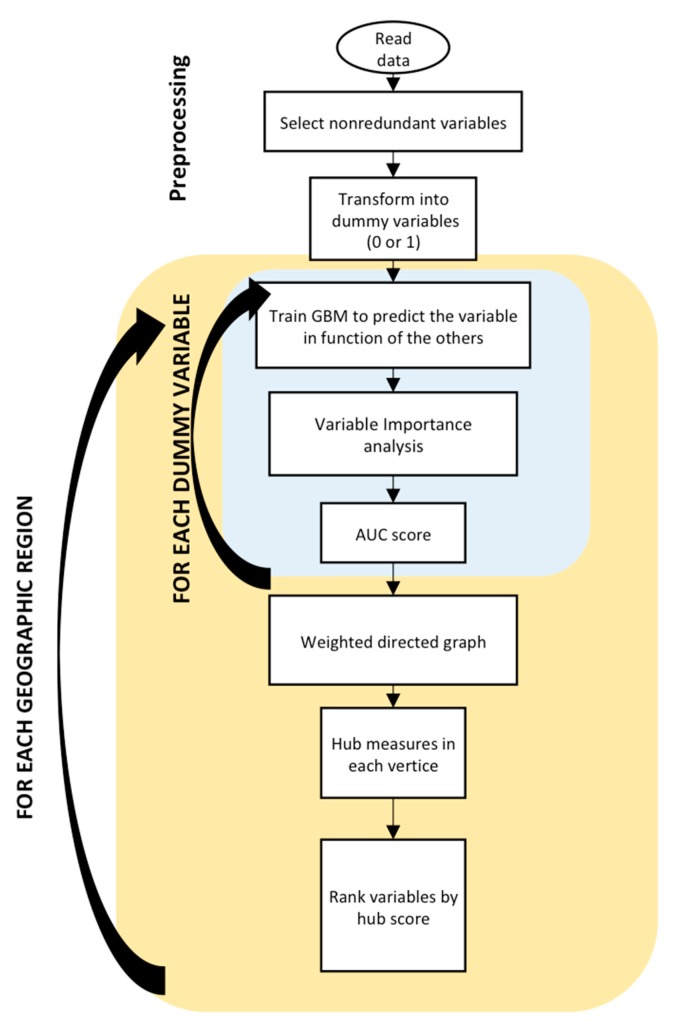
Flowchart illustrating the procedures and the steps carried out. GBM—gradient boosting machines and AUC—area under the receiver operator characteristic curve.

A flowchart illustrating the general procedure is shown in Figure 1. First, the PeNSE dataset was loaded and preprocessed, as described in the Data Preprocessing section. Then, the dataset was split according to Brazil’s five geopolitical regions, which are well-established spatial clusters of the country (North, Northeast, Central-West, South, and Southeast). These five regions are well-established in Brazil, since they have several heterogeneous features (sociodemographic, economics, ethnicity, climate, natural resources, etc.). Thus, the results of our analyses could potentially be highly different among these five regions. For each region, a predictive model for a chosen target variable was built based on GBM, using the other variables as predictors. For each predictive model, the importance of the predictor variables (from the training data) and area under the receiver-operating characteristic curve (AUC of test data) was then calculated. A directed weighted graph (network) was modeled based on the yielded variables importance. Then, graph analyses were applied to identify the main hubs of this network (i.e., the main potential confounders). Finally, the similarity of the findings across the five regions was investigated.

### 2.3. Data Preprocessing

Since all variables included in this analysis were categorical (see Supplementary Material for a translated description), they were transformed into dummy variables (one-hot encoding) to consider multiclass categorical variables as predictors and also to homogenize all predictive models in a two-class classifier. The data preprocessing generated a total of 639 binary variables to be analyzed. To avoid data leakage (as a consequence of one hot encoding step), the dummy variables generated from the same original variable were not considered as predictor variables in any model in which they were the target variables.

### 2.4. Gradient Boosting Machines

Gradient boosting is a machine learning technique that combines the outcomes of several shallow decision trees to produce a rather robust predictive model [10]. Decision trees are models that, aiming to estimate a target variable, recursively split the available dimensions (features) of a given dataset into binary partitions [5]. In a classification problem, the splits are chosen with the goal of maximizing the purity in the resulting partitions (i.e., the proportion of a target’s class with respect to the others). By combining several decision trees in particular manners (e.g., bagging or boosting), it is possible to achieve a greater prediction performance [11,12]. Technical details on the boosting method can be found in algorithm 6, in [13].

For each geographic region, we have split the data by 70% for training data and 30% for testing. In the current study, the AUC (area under the receiver-operating characteristic curve) of the test data was considered as the accuracy metric for the predictions. The extraction of the importance (contribution) of each variable in a trained GBM was conducted using the method proposed by [13], based on the “gain” of each variable, considering the partitions of the trees composing the boosted ensemble. The metric quantifies the squared error reduction due to the split at the referred variable. For classification problems, the gain is associated with the increase in purity after a split, due to a given feature. Thus, the relative feature importance for gradient boosting is the average purity increase over all sequential trees.

### 2.5. Graph Analysis

Networks can be used to illustrate the relations between entities. In this study, we modeled a network to illustrate which variables explain other variables in a machine learning model. In this network, the nodes were the variables of interest and the edges were the predictive importance between the variables. Then, we identified the most central variables in the network using the hub score metric. This centrality measure could be interpreted as a metric of how much information a node “sends” to the other nodes of the network. In the context of the current study, this means that one variable with a high hub score is important as a predictor in several GBM models. In other words, the hub score determined which variables had the most predictive information regarding the other variables in the dataset. Thus, the variables with high hub scores can be interpreted as potential confounders or at least variables that should be taken into account in association analyses. To obtain the hub score, we used the method proposed by [14]. Instead of simply summing up the outgoing edges, which is an analysis from a local perspective, the hub centrality also considers the neighbors of the neighbors (global analyses). This was obtained as the principal eigenvector of A×A^T^, in which A is defined as the graph adjacency matrix of the network, and A^T^ is its transpose. The adjacency matrix used for the hub score computation was an N×N matrix, N being the number of nodes in the graph, in which the rows represent the outgoing links, and the columns represent the incoming links.

### 2.6. Predictive Networks

In our study, we applied the concept of a predictive network, in which a predictive model for each variable was built using the other variables as predictors. The predictive models were built using the GBMs, which can quantify the importance of each variable in this model (100 trees, max depth of 4, implemented in h2o package, www.h2o.ai) using the method described at Section 2.4. By using these variable importance values (which are positive continuous metrics), we built an adjacency matrix for modeling a directed weighted graph. No thresholds were applied to these edges. Then, we analyzed the graph to identify the main hubs of the network (e.g., the variables which were the most important to predict the other variables), implemented using the igraph package [15]. All analyses were carried out in R platform version 3.5.0 for Computational Statistics (www.r-project.org).

This same procedure was repeated for each of the five independent geographical regions of the country in order to check results replicability and similarities across regions. This analysis was important to demonstrate that the identified network hubs were not obtained by chance.

## 3. Results

**Figure 2:**
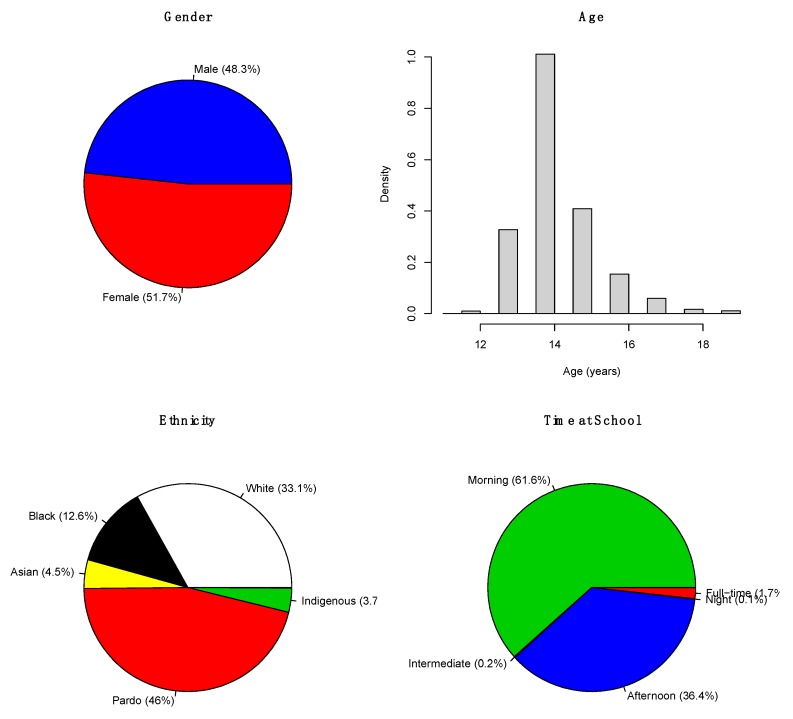
Demographical information.

Figure 2 presents a demographical characterization of our sample. Most of the adolescents were 14-years old (since they were at the same grade level), gender was well-balanced (48.3% males), and 61.6% of the participants attended the classes during the morning. 

**Figure 3:**
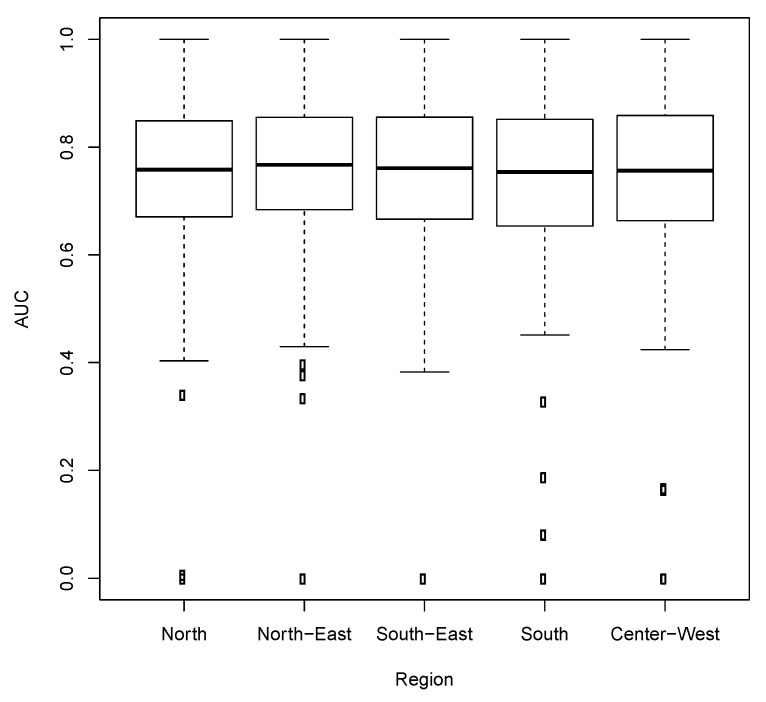
Boxplot for the area-under-the-curve (ROC) across variables for each geographic region.

Since our proposal was based on fitting on GBM for each binary variable, each fitted model had a prediction accuracy quantified by the area under the AUC obtained in the test data. Figure 3 highlights that the AUC distribution (across the 639 variables) was very homogeneous among the five-country regions. Moreover, the AUCs were moderately high with a median between 0.7 and 0.8 and with a distribution far away from 0.5 (chance level).

**Figure 4:**
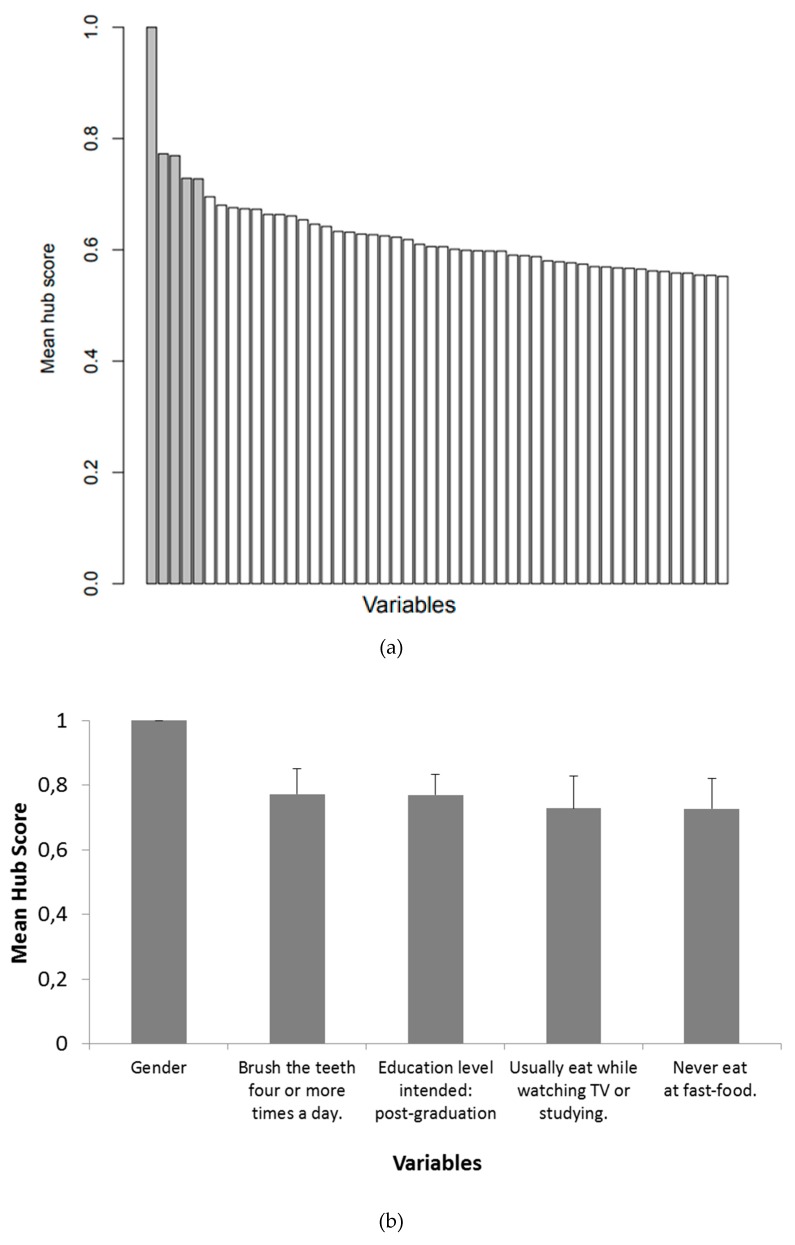
Mean hub scores (across regions) for each variable (**a**) and a detailed zoom at the top-5 ones ((**b**), with standard deviation across regions). For visualization purposes of hub score decay, only the first 50 top ranked variables are shown (at top).

The core analysis of the current study is depicted in Figure 4. Figure 4 (top) depicts the decay of the hub score, highlighting that the mean hub score was a continuous measure. Regarding the parsimony of interpretation and discussion, we only considered the top 5 ranked variables as the main potential confounder variables of the PENSE dataset. Figure 4 (bottom) highlights that gender was the top hub score from the variables network, meaning that it was important to predict most of the other variables in the dataset. In addition, although not outstanding as gender, “brushing the teeth 4 or more times a day”, “post-graduation” as the intended education level, “usually eat when watching TV or studying”, and “never eat fast-food” were at the top 5 ranked variables when considering the hub score.

**Figure 5:**
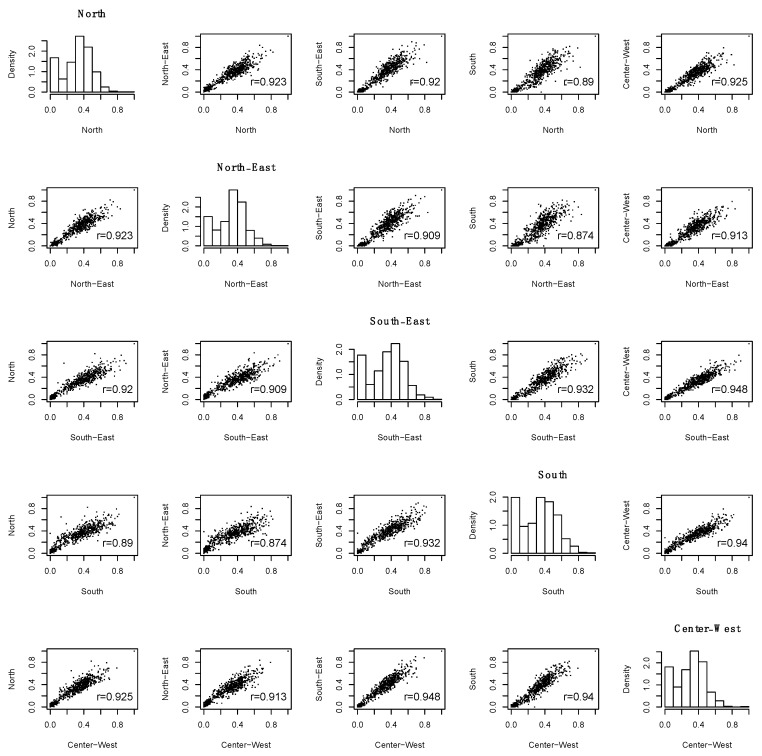
Histogram, scatter-plot, and Pearson correlation coefficient of the hub scores of each variable across the five geographic regions.

On the contrary, it was important to investigate how the hub scores varied across the five Brazilian main geographical regions. If the scores were completely different, they could suggest that the regions were very heterogeneous on the variable’s covariance structure or that the proposed approach was inconsistent. However, Figure 5 demonstrates that the variables’ hub score were indeed very similar across regions, with very high Pearson correlation coefficients and linear relation. This finding suggests that the covariance structure among the variables was similar despite regional differences and that our results are replicable in independent datasets (and thus, the results were not obtained by chance).

## 4. Discussion

In the current study, we aimed to obtain insights into potential confounders in an adolescent public health dataset of a developing country, by using a combination of machine learning methods and graph analysis. The main strength of the method is that the analyses were conducted from a multivariate perspective (i.e., combining different variables) and the assessment was regarding the predictive relevance of each variable. By using a combination of gradient boosting machines and centrality hub metric, it was possible to evaluate the most informative variables to predict the others, from a network perspective. As highlighted in the Introduction, the identification of these hubs is relevant to unveil potential confounders to be taken into account. As expected, variables related to gender and nutrition were found to be the most informative regarding adolescence health in Brazil.

It is important to mention that, in all five-country regions, the median AUC across the 639 variables was greater than 0.75 (and the first-quartile nearly 0.7). This finding reinforces that gradient boosting machinesgradient boosting machines are indeed capable of capturing the dependence structure among the variables to build predictive models. Since the redundant variables were removed from the input set, the third-quartile of the AUC was between 0.8 and 0.9 for all regions. In addition, it is noteworthy that the distribution of AUC was similar across geographical regions. Moreover, the hub metrics derived from the predictive networks were also very similar in their distribution and replicability across regions.

Gender was found to be the top-ranked predictor in all regions (i.e., the main potential confounder). Thus, our findings suggest that adolescent public health analysis should take into account gender differences not only regarding physiological features, but also in nutrition, perception, and behavior.

Interestingly, oral health care was the second-ranked predictor. Specifically, the variable of whether the participant brushed his/her teeth more than four times a day was at the top-five. Associations between oral health and cardiovascular diseases have been reported in the literature [16,17]. Nevertheless, this relation might not be causal, and this phenomenon is still not elucidated. A recent study based on the PenSE database [18] indicated that in fact the prevalence of simultaneous oral health-risk behaviors is associated with sociodemographic factors—family supervision is highlighted as a protective factor for multiple oral health-risk behaviors, which adds evidence to the importance of family’s role in an adolescents’ health-related choices. Although the mechanistic explanation for this association is still under debate, it reinforces that oral health self-assessment might play a role as a proxy variable for other health conditions. Finally, the variable “brushing the teeth more than four times a day” in our findings mirrors a high standard of self-care in Latin America [19]. We speculate that this standard of self-care is also extrapolated to other variables related to general health.

Nutrition condition is a natural candidate for source of predictive information regarding general health, and we had previously hypothesized that nutrition-related variables would be ranked within the top-5. However, in our findings, the variables “usually eat when watching TV or studying” and “never eat at a fast-food establishment” might reflect a myriad of other conditions beyond nutrition. First, “eating when watching TV or studying” might be a proxy for family daily functioning, socio-economic status, self-awareness, and even coping with adverse situations. Similarly, “eating at a fast-food establishment” might refer to socio-economic status. Similarly, the presence of “intended educational level” at the top-five is also interesting. We expected that parents’ education would be a more relevant variable, which is traditionally associated with socio-economic status. However, it is important to have in mind that the questionnaires were filled by adolescents, who might not precisely know about their parents’ education. Moreover, the intended education level might be considered as a combination of the parental and student perceptions and perspectives.

Thus, analogous to the oral health conditions and gender, the nutrition variables are also a proxy for many other variables. It is important to mention that the results were obtained in a completely data-driven approach. Moreover, Figure 3, Figure 4 and Figure 5 emphasize that the hub centrality rank is replicable across the five-country regions with slight differences, thus, these findings seem to be robust and not obtained by chance.

It is important to mention that our study presents relevant limitations. First, causal effects cannot be inferred using the proposed approach, and thus, the predictive networks are not causal networks. Second, although beyond the scope of our study, the comparison between different machine learning methods and other graph analysis metrics could also provide further insights. However, we expect that future studies might profit from our outcomes and strategies to improve the survey instruments and analysis related to public health. For example, the identification of the confounders and relevant variables could be exploited to reduce data dimensionality and improve the efficacy of propensity score matching or other analytical tools.

In conclusion, we believe that the proposed approach based on predictive networks is a promising tool to obtain novel insights on the association between variables and to identify general factors related to health conditions, which could be considered as potential confounders.

## Supplementary Materials

The following are available online at https://www.mdpi.com/1660-4601/17/1/90/s1: Translated description.
